# Genetic inhibitors of APOBEC3B-induced mutagenesis

**DOI:** 10.1101/gr.277430.122

**Published:** 2023-09

**Authors:** Tony M. Mertz, Elizabeth Rice-Reynolds, Ly Nguyen, Anna Wood, Cameron Cordero, Nicholas Bray, Victoria Harcy, Rudri K. Vyas, Debra Mitchell, Kirill Lobachev, Steven A. Roberts

**Affiliations:** 1 School of Molecular Biosciences and Center for Reproductive Biology, Washington State University, Pullman, Washington 99164, USA;; 2 Department of Microbiology and Molecular Genetics, University of Vermont Cancer Center, University of Vermont, Burlington, Vermont 05405, USA;; 3 School of Biological Sciences, Georgia Institute of Technology, Atlanta, Georgia 30332, USA

## Abstract

The cytidine deaminases APOBEC3A (A3A) and APOBEC3B (A3B) are prominent mutators of human cancer genomes. However, tumor-specific genetic modulators of APOBEC-induced mutagenesis are poorly defined. Here, we used a screen to identify 61 gene deletions that increase A3B-induced mutations in yeast. We also determined whether each deletion was epistatic with Ung1 loss, which indicated whether the encoded factors participate in the homologous recombination (HR)–dependent bypass of A3B/Ung1-dependent abasic sites or suppress A3B-catalyzed deamination by protecting against aberrant formation of single-stranded DNA (ssDNA). We found that the mutation spectra of A3B-induced mutations revealed genotype-specific patterns of strand-specific ssDNA formation and nucleotide incorporation across APOBEC-induced lesions. Combining these three metrics, we were able to establish a multifactorial signature of APOBEC-induced mutations specific to (1) failure to remove H3K56 acetylation, (2) defective CTF18–RFC complex function, and (3) defective HR-mediated bypass of APOBEC-induced lesions. We extended these results by analyzing mutation data for human tumors and found BRCA1/2-deficient breast cancers display three- to fourfold more APOBEC-induced mutations. Mirroring our results in yeast, Rev1-mediated C-to-G substitutions are mainly responsible for increased APOBEC-signature mutations in BRCA1/2-deficient tumors, and these mutations associate with lagging strand synthesis during replication. These results identify important factors that influence DNA replication dynamics and likely the abundance of APOBEC-induced mutation during tumor progression. They also highlight a novel role for BRCA1/2 during HR-dependent lesion bypass of APOBEC-induced lesions during cancer cell replication.

Tumorigenesis is driven by mutations in oncogenes and tumor-suppressor genes. Additional mutations allow tumors to grow, metastasize, and develop resistance to chemotherapeutics. Large-scale whole-genome sequencing of tumors has identified 81 mutation signatures as indexed by the Catalog of Somatic Mutations in Cancer (COSMIC) (Alexandrov et al. 2020) that represent processes mediating tumor genome evolution such as endogenous and exogenous sources of DNA damage, defects in DNA repair, or decreased DNA replication fidelity (Lynch and de la Chapelle 2003; Imai and Yamamoto 2008; The Cancer Genome Atlas Network 2012; The Cancer Genome Atlas Research Network 2013; Palles et al. 2013; Alexandrov et al. 2020). However, the mechanisms that generate many mutation signatures are poorly understood or unknown.

Single-base-pair substitution (SBS) signature 2 and SBS13 (Alexandrov et al. 2020) are primarily composed of C-to-T or C-to-G mutations, respectively, within TCW trinucleotide motifs (with W being either an A or T). These two mutation signatures are second only to aging-signature mutations in their contribution to mutation burden in cancer (Alexandrov et al. 2013), are overrepresented in ∼30% of all sequenced tumors (Alexandrov et al. 2020), and, in many tumors, constitute >50% of all mutations (Burns et al. 2013b; Roberts et al. 2013). These mutation signatures have been attributed to APOBEC cytidine deaminases, which normally play diverse biological roles in the innate and adaptive immune responses (Refsland and Harris 2013). When dysregulated, APOBECs mutate genomic DNA by converting deoxycytidine (dC) to deoxyuridine (dU) within regions of single-stranded DNA (ssDNA), primarily during DNA replication but also during transcription and DNA repair (Chaudhuri et al. 2003; Nik-Zainal et al. 2012; Roberts et al. 2012; Lada et al. 2013; Kazanov et al. 2015; Haradhvala et al. 2016; Hoopes et al. 2016). Multiple lines of evidence all indicate A3A and A3B are primarily responsible for the large number of C-to-T or C-to-G mutations within TCW motifs found in cancer genomes (Harris et al. 2002, 2003; Bishop et al. 2004; Yu et al. 2004; Bogerd et al. 2006; Dang et al. 2006; Henry et al. 2009; Lackey et al. 2013; Burns et al. 2013a,b; Roberts et al. 2013; Hoopes et al. 2016; Adolph et al. 2017; Cortez et al. 2019; Petljak et al. 2022).

APOBEC activity causes both primary driver mutations and late subclonal driver mutations in cancer (de Bruin et al. 2014; Henderson et al. 2014; McGranahan et al. 2015; The Cancer Genome Atlas Research Network 2017; Jamal-Hanjani et al. 2017). In addition, APOBEC expression and APOBEC signature mutations have prognostic value for patient outcomes and responses to cancer therapeutics (Sieuwerts et al. 2014; Walker et al. 2015; Billingsley et al. 2016; D'Antonio et al. 2016; Law et al. 2016; Middlebrooks et al. 2016; Yan et al. 2016; Glaser et al. 2018; Van Hoeck et al. 2019; Alexandrov et al. 2020). Because of these links to cancer progression and survival, several studies have characterized defects that reduce cell viability when combined with APOBEC expression (Mehta et al. 2020; Biayna et al. 2021). In contrast, the modulators of APOBEC-induced mutagenesis in tumor cells are largely unknown. A3A and A3B mRNA expression correlates with the number of APOBEC-induced mutations in tumors. However, these correlations are generally weak, which suggests that in addition to transcriptional dysregulation, multiple factors such as APOBEC protein stability, post-translational modifications, substrate availability, and repair of APOBEC-induced dU likely play significant roles in modulating APOBEC-induced mutagenesis in tumors. In this study, we used a yeast screen to identify genetic defects that increase APOBEC-induced mutagenesis and subsequently determined what mechanistic gene functions and processes were likely responsible. We also conducted bioinformatic analyses to determine if processes that protect against APOBEC-induced mutagenesis in our yeast model system function similarly in human tumors.

## Results

**Figure 1.**
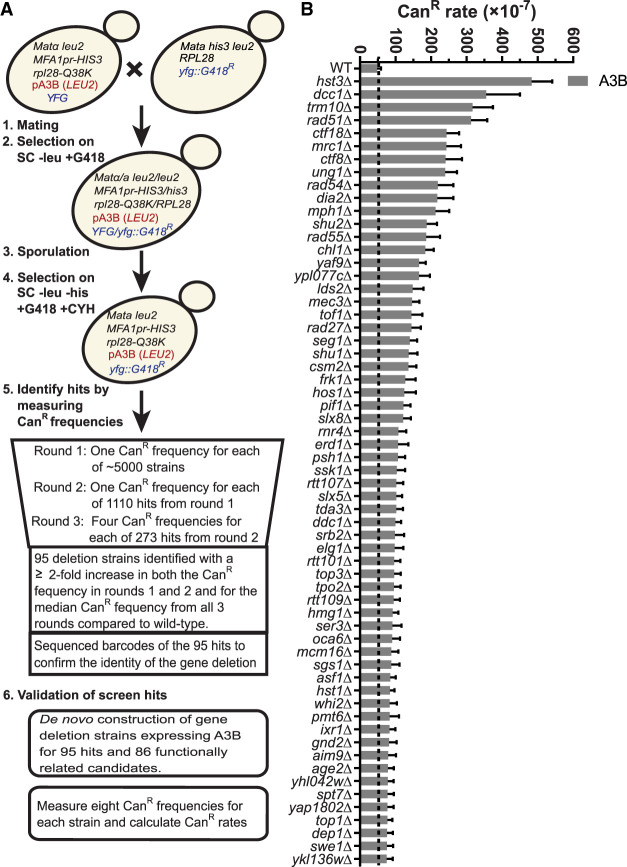
Identification of yeast gene deletions that increase APOBEC3B-induced mutations. (*A*) Schematic of the yeast screen used to identify gene deletions that elevate A3B-induced mutagenesis. Haploid yeast containing a single-gene deletion and the A3B expression plasmid was obtained by mating *MAT*α yeast carrying the A3B expression plasmid with each *MAT*a strain of the yeast gene deletion library, sporulating the resulting diploid cells, and selecting the desired haploid genotype. The resulting haploid cells were used for three rounds of Can^R^ frequency measurements to identify gene deletions that likely augment A3B-induced mutagenesis. We were unable to generate 21 haploid strains for the screening process that represent potential (unvalidated) synthetic lethal interactions with A3B expression (Supplemental Table S1). To confirm these deletions resulted in increased A3B-induced mutations we recreated each deletion strain expressing A3B de novo and measured Can^R^ mutation rates. (*B*) Can^R^ rates for 61 yeast gene deletions that elevate A3B-induced mutation more than 1.55-fold over the A3B-induced Can^R^ rate in wild-type yeast (gray dashed line) without overlapping 95% confidence intervals and 1.55-fold higher than additive for spontaneous deletion-dependent Can^R^ and wild type with A3B Can^R^ rates. Error bars indicate 95% confidence intervals. For all mutation rate data, see Supplemental Table S2.

Because of the difficulty of measuring mutagenesis in cultured human cells, we used *Saccharomyces cerevisiae* as a model system to screen for genes preventing APOBEC-induced mutagenesis. We created a library of haploid yeast deletion strains expressing A3B using a mating, sporulation, and selection strategy (Fig. 1A). We identified ORF deletions (screen hits) that increased the frequency of canavanine-resistant (Can^R^) colonies at least twofold in each of three successive rounds of measurements (Supplemental Table S1). This set of hits contained most of the gene deletions previously shown to increase APOBEC-induced mutations, including *ung1*Δ, *mph1*Δ, and *tof1*Δ (Hoopes et al. 2016, 2017), supporting the effectiveness of the screening strategy. Based on the known functions and interactors of the screen hits, we also created a list of additional candidate genes with probable roles in restricting APOBEC-induced mutagenesis (see Supplemental Table S1). To validate whether the screen hits and candidates increased A3B-induced mutation, we transformed either haploid strains from the BY4741 yeast deletion library (i.e., not created via mating/sporulation) or de novo deletion strains constructed from the wild-type BY4741 yeast with an A3B expression plasmid (and separately an empty vector control) and measured *CAN1* mutation rates (Supplemental Table S2). Gene deletions present in yeast strains that met the following metrics were classified as bona fide modulators of APOBEC-induced mutagenesis: (1) 95% confidence intervals for *CAN1* mutation rates were nonoverlapping between the wild-type and deletion strains expressing A3B; (2) the A3B-induced mutation rate was more than 1.55-fold higher in the deletion strain compared with the wild type; and (3) the A3B-induced mutation rate was more than 1.55-fold higher than the sum of the spontaneous Can^R^ rate (for that strain) and the rate for the wild-type strain with A3B expression, which ensures the increase in the mutation rate is owing to elevated A3B-induced mutagenesis and is not an additive effect for strains with a relatively high spontaneous mutation rate (Supplemental Table S2). In total, we identified 61 gene deletions that increase A3B-induced mutagenesis (Fig. 1B). Deletion of *HST3*, members of the Ctf18–RFC complex, *TRM10*, *MRC1*, homologous recombination (HR) factors, and *UNG1* were among those with the greatest increase in A3B-induced mutation. To determine if A3B-induced mutagenesis resulted from changes in A3B expression, we performed RT-qPCR on a subset of the yeast deletion strains with the A3B expression plasmid (30 total) and calculated fold changes in A3B expression using the wild-type strain with A3B for comparison (Supplemental Table S3). Of these 30 strains, only *psh1*Δ and *srb2*Δ displayed an increase in A3B expression that could explain the A3B-induced *CAN1* mutation rates observed.

**Figure 2.**
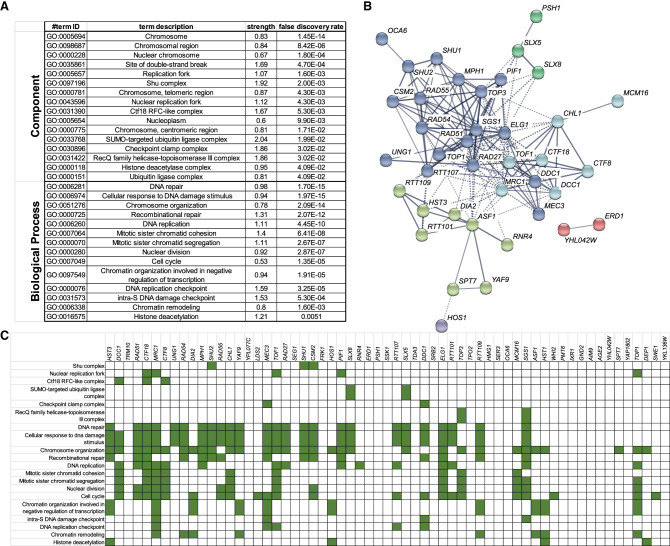
Molecular processes enriched among gene deletions that increase A3B-induced mutagenesis. (*A*) Gene Ontology (GO) analysis for molecular processes that are enriched among gene deletions that increase A3B-induced mutation. Only categories with strengths equal to or greater than one and having at least three genes represented in the category are shown. Categories for similar processes are represented with the specific GO process with the lowest FDR-corrected *P*-value. (*B*) Functional association network for proteins that limit A3B-induced mutation as created by STRINGdb. The 61 gene deletion strains that were confirmed to increase A3B-induced mutagenesis were used in creating the network, with highest-confidence linkage settings and *k*-means clustering with eight nodes. Proteins without connections to other proteins in the network are hidden. Each *k*-means clustering node is indicated by a unique color. (*C*) Gene deletions that elevate A3B-induced mutation function in multiple GO processes. The specific enriched GO processes that each gene contributes to are indicated in green.

Gene Ontology (GO) analysis of the 61 validated defects that increased A3B-induced mutagenesis indicated this gene set was highly enriched for genes with many functions in DNA metabolism (Fig. 2A). Analysis of interactions between these genes via STRINGdb indicates that HR, histone H3 lysine 56 acetylation (H3K56ac), and the CTF18–RFC complex likely play significant roles in APOBEC-induced mutagenesis (Fig. 2B). However, numerous interactions between genes of different clusters and our observation that many genes within this data set are associated with multiple complexes and/or functional processes (Fig. 2C) made it difficult to determine which function(s) of individual genes act to limit APOBEC-generated mutations.

**Figure 3.**
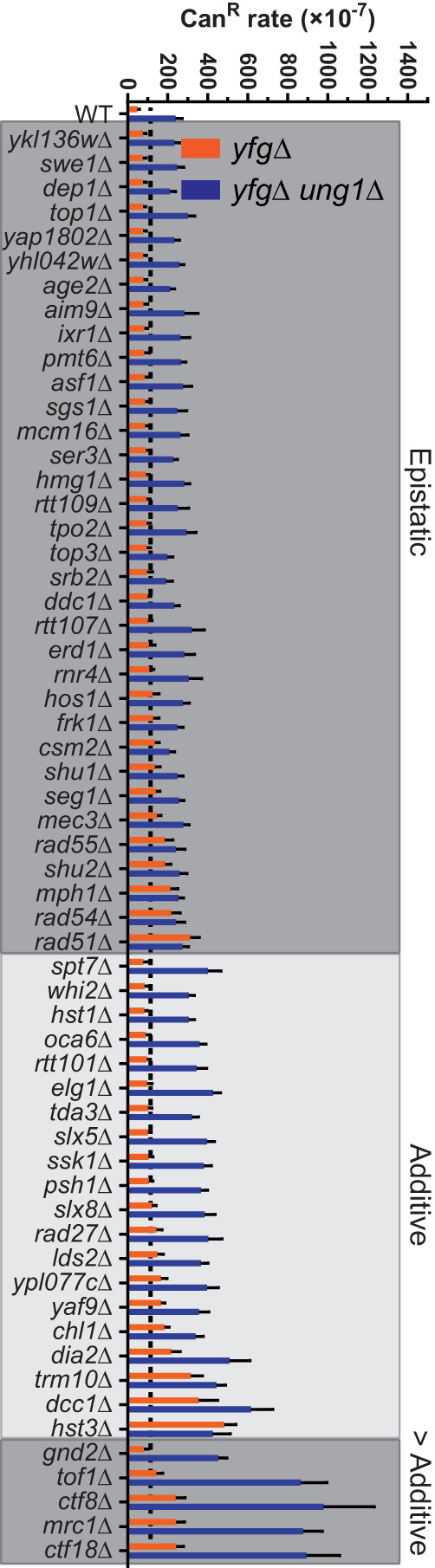
Impact of *ung1*Δ in mutants showing increased A3B-induced mutation. A3B-induced Can^R^ rates in yeast that contains genes that limit A3B-induced mutation (*yfg*Δ; orange bars) or in yeast with genes that limit A3B-induced mutation codeleted with *UNG1* (*yfg*Δ *ung1*Δ; blue bars). Error bars indicated 95% confidence intervals. Double-deletion strains (*yfg*Δ *ung1*Δ) that maintain similar A3B-induced Can^R^ rates to the single-deletion strain and *ung1*Δ strains (black dashed line) are considered epistatic with *ung1*Δ and likely contribute to the repair or error-free bypass of A3B-induced lesions. *yfg*Δ *ung1*Δ strains with A3B-induced Can^R^ rates higher than *ung1*Δ strains are either additive or greater than additive with *UNG1* deletion and likely increase the amount of ssDNA that A3B acts upon.

In yeast (Hoopes et al. 2016) and human cells (Haradhvala et al. 2016), the primary source of APOBEC signature mutations is deamination of dC present on the lagging strand template during DNA replication. The resulting dUs in this context can be converted to an abasic site by a uracil DNA glycosylase: Ung1 in yeast or UNG2 in human cells. Alternatively, unremoved dUs template for DNA synthesis, which always results in a C-to-T mutation because dU templates like deoxythymidine. Neither mismatch repair or base excision repair (BER) can prevent mutations resulting from dU:dA base pairs, which is consistent with our findings that prevention of APOBEC-induced mutations requires Ung1 and that in *ung1*Δ strains all APOBEC-induced dUs result in C-to-T mutations (Hoopes et al. 2017). Because all APOBEC-induced dUs are mutagenic in *ung1*Δ strains, defects that significantly increase APOBEC-induced *CAN1* mutation rates above *ung1*Δ alone likely increase APOBEC-induced dUs by increasing the amount of ssDNA available to serve as substrate for APOBEC activity. BER cannot be completed within ssDNA; therefore, Ung1-generated abasic sites must either be bypassed by translesion synthesis (TLS) or HR-dependent lesion bypass to allow for completion of DNA replication. Deletions that are epistatic to *ung1*Δ for *CAN1* mutation rates in the presence of A3B likely function during HR-dependent bypass of abasic sites created by combined activities of A3B and Ung1 (Supplemental Fig. S1A). Therefore, we combined the yeast deletions that increased APOBEC-induced mutagenesis with *ung1*Δ, expressed A3B in these strains, and measured mutation rates (Fig. 3). Supporting previous findings, we found that deletions of *MPH1* (Hoopes et al. 2017) and Shu-complex members *CSM2*, *SHU1*, and *SHU2* (Rosenbaum et al. 2019), genes previously shown to be important for HR-mediated lesion bypass (Godin et al. 2016), were epistatic to *ung1*Δ for A3B-induced mutagenesis. We also found epistasis between *ung1*Δ and deletions of genes encoding proteins with direct roles in HR (i.e., *RAD51*, *RAD54*, and *RAD55*) and recombination-intermediate resolution (i.e., *SGS1* and *TOP3*) (Fabre et al. 2002). Deletion of *MEC3* and *DDC1*, which are members of the yeast 9-1-1 complex, was also epistatic with *ung1*Δ supporting works, suggesting that they have a role in HR-dependent lesion bypass (Karras et al. 2013). Conversely, the deletion of components of replication checkpoint surveillance complex, *MRC1* and *TOF1*, greatly increased the rate of A3B-induced mutagenesis when combined with *ung1*Δ, consistent with their roles at stalled replication forks (Katou et al. 2003; Tourrière et al. 2005; Hodgson et al. 2007). Also, the deletion of *CTF18* and *CTF8*, which are members of the CTF18–RCF complex, which has multiple roles in DNA replication and repair (Mayer et al. 2001; Bylund and Burgers 2005; Ogiwara et al. 2007; Garcia-Rodríguez et al. 2015), when combined with *ung1*Δ produced a rate of A3B-induced mutagenesis much greater than that of either single deletion alone, which suggests that at least one function of CTF18–RFC reduces levels of ssDNA.

**Figure 4.**
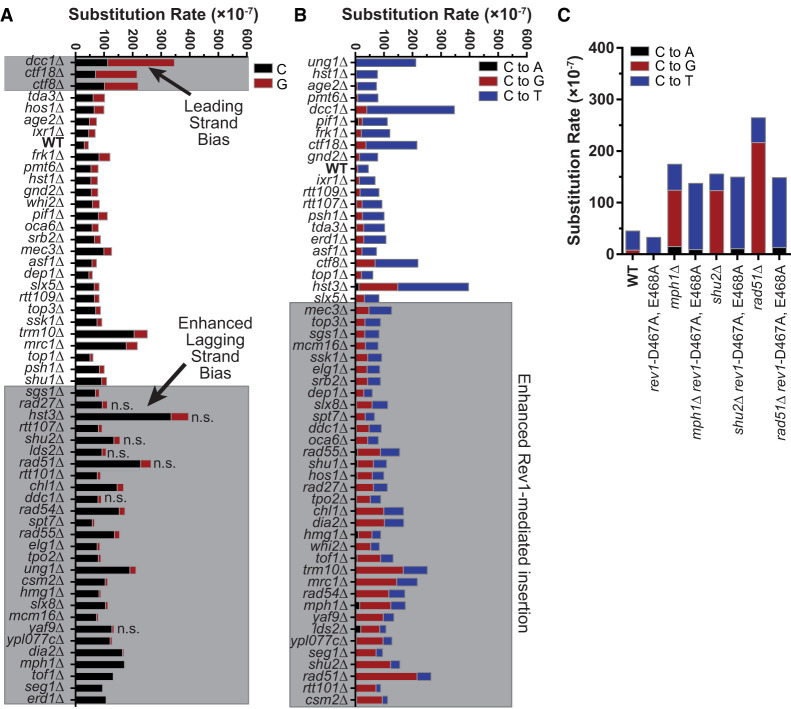
Spectra of A3B-induced mutations in *CAN1.* The *CAN1* gene of independent Can^R^ isolates was amplified and sequenced to determine the spectra of A3B-induced mutations in yeast with specific gene deletions. Note that A3B-induced *CAN1* mutants were not sequenced from the *aim9*Δ, *rnr4*Δ, *ser3*Δ, *swe1*Δ, *yap1802*Δ, *YKL136W*Δ, and *YHL042W*Δ strains. (*A*) Strand bias of A3B-induced mutations in *CAN1* is influenced by specific gene deletions. The rate of C (black bars) or G (red bars) substitutions in each genotype of yeast expressing A3B. Wild-type yeast expressing A3B favors C substitutions over G substitutions, indicating that the lagging strand template is the top DNA strand of *CAN1* at its location on ChrV. The ratio of A3B-induced C-to-G substitutions in wild-type yeast was compared to the same ratio for each yeast deletion strain by two-sided Fisher's exact test. Genotypes with *P*-values < 0.00083 (based on Bonferroni multiple hypothesis testing correction) were considered to have altered strand bias. (*B*) The rates of C-to-A, C-to-T, and C-to-G substitutions (complementary substitutions were combined) in *CAN1* for yeast expressing A3B and containing various gene deletions. The ratio of C-to-T and C-to-G substitutions were compared pairwise between wild-type yeast expressing A3B and each specific gene deletion strain by two-sided Fisher's exact test. Gene deletion strains displaying *P*-values < 0.00083 (for Bonferroni correction) were deemed to have more A3B-induced abasic sites bypassed via Rev1 catalytic activity. (*C*) Rates of C-to-A, C-to-T, and C-to-G substitutions (as in *B*) for comparing WT and recombination-deficient yeast with corresponding strains lacking Rev1 catalytic activity (i.e., *rev1*-D467A, E468A).

To gain additional insight as to how individual gene deletions increase A3B-induced mutagenesis, we sequenced *CAN1* mutants from each deletion strain expressing A3B. Consistent with our conclusion that the identified genes protect against APOBEC-induced mutations, >70% of mutations for each genotype expressing A3B involved C-to-G or C-to-T substitutions at TC dinucleotides (83% for all genotypes combined) and therefore are attributable to A3B activity (Fig. 4; Supplemental Tables S4, S5). We and others have shown that APOBECs primary target the lagging strand template during DNA replication (Bhagwat et al. 2016; Haradhvala et al. 2016; Hoopes et al. 2016; Morganella et al. 2016; Seplyarskiy et al. 2016; Saini et al. 2017; Sui et al. 2020). In wild-type BY4741 yeast with A3B expression, the lagging strand template is associated with 2.1-fold more APOBEC-induced mutations for the *CAN1* coding sequence (C nucleotides are favored over G nucleotides at *CAN1* in this genomic location) (Supplemental Fig. S1A). Like *ung1*Δ, many deletions of genes with likely roles in HR-dependent lesion bypass (*sgs1*Δ, *rad54*Δ, *rad55*Δ, *csm2*Δ, and *mph1*Δ) had a significant increase in mutations at C nucleotides, whereas others (*shu2*Δ and *rad51*Δ) had a similar, but not statistically significant, increase (Fig. 4A), indicating loss of HR-dependent lesion bypass emphasizes the lagging strand template bias of A3B-induced mutation. Conversely, defects in all three members of the CTF18–RCF complex, *ctf18*Δ, *ctf8*Δ, and *dcc*1Δ (Fig. 4A), resulted in a leading strand template mutation bias indicative of increased ssDNA on the leading strand template.

We also analyzed the *CAN1* mutation spectra to assess if gene deletions affected the ratio of C-to-G to C-to-T A3B-induced mutations with higher ratios indicating increased Rev1-mediated TLS to bypass A3B-dependent abasic sites and lower C-to-G to C-to-T ratios indicating enhanced A-rule bypass or decreased Ung1 activity (Fig. 4B). As expected, in *ung1*Δ stains, only A3B-induced C-to-T substitutions were observed. Deletions causing defects in HR-dependent lesion bypass significantly increased the C-to-G to C-to-T ratio, and the increase of the *CAN1* mutation rate in these strains is almost entirely owing to increased C-to-G mutations. To determine if the increased C-to-G to C-to-T ratio in HR-dependent bypass-deficient strains is owing to increased usage of TLS that uses Rev1, we measured mutation rates (Supplemental Table S2) and produced mutation spectra (Supplemental Table S4) in strains with a *rev1-D467A, E468A* allele, which encodes a Rev1 variant that lacks deoxycytidyl transferase activity but can still serve as a scaffold to support TLS function (Nelson et al. 1996; Guo et al. 2003, 2006; Zhou et al. 2010). We found that the *rev1-D467A, E468A* mutation did not significantly change *CAN1* mutation rate in the wild-type, *rad51*, *mph1*, and *shu2* yeast strains. However, nearly all the mutations in strains with the *rev1-D467A, E468A* mutation were C-to-T in APOBEC target motifs (Fig. 4C). Together, these results suggest that A3B-dependent abasic sites that are not bypassed by HR-dependent template switching are primarily bypassed using Rev1 deoxycytidyl transferase activity and, when this activity is absent, that use of an A-rule polymerase for bypass results in a C-to-T mutation. In addition, elevated A3B mutagenesis in the *dia2*Δ, *mrc1*Δ, and *tof1*Δ stains was primarily driven by increased C-to-G mutations, indicating that maintenance of replication fork stability normally suppresses A3B-induced abasic sites bypassed by Rev1-dependent TLS. Deletion of factors influencing H3 K56 acetylation (i.e., *HST3*, *RTT107*, *ASF1*) and disruption of the CTF18–RCF complex (*ctf18*Δ, *ctf8*Δ, or *dcc*1Δ), however, increased the rate of both C-to-G and C-to-T substitution.

**Figure 5.**
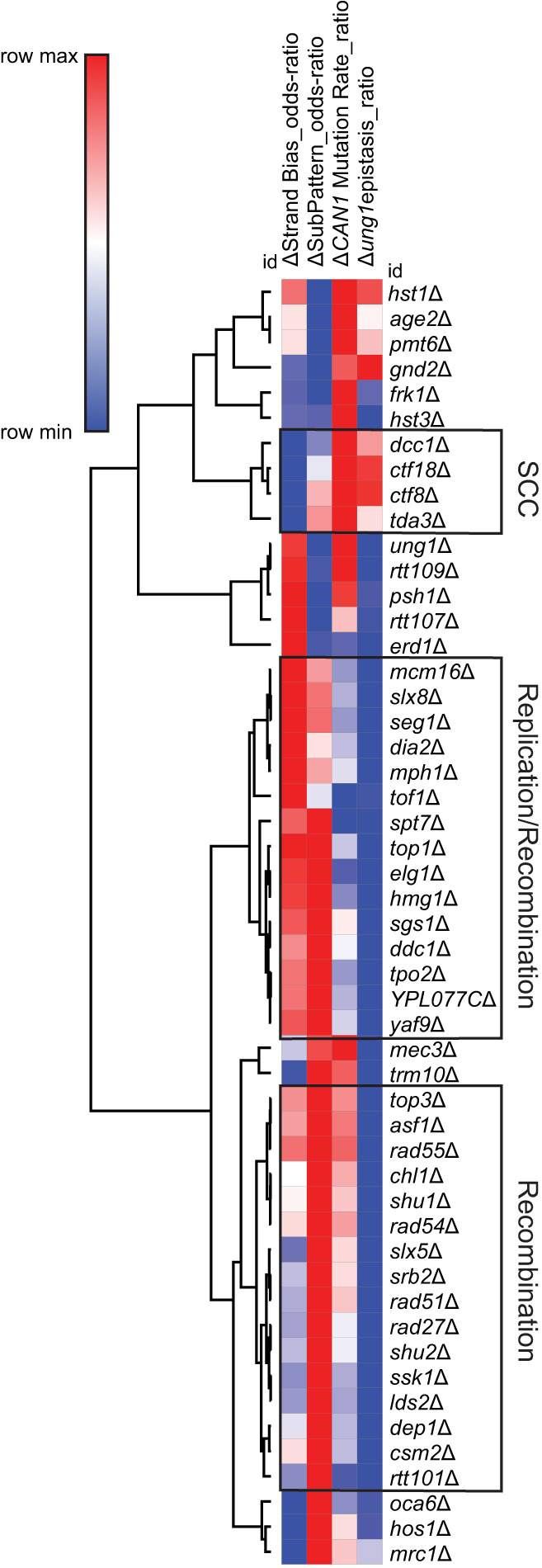
Nonbiased hierarchical clustering of yeast gene deletions that similarly impact A3B-induced mutation. Yeast gene deletion strains with greater than 20 independent A3B-induced *CAN1* mutations sequenced were clustered using average linkage based upon (1) how much the gene deletion elevated A3B-induced mutation over that in wild-type yeast, ΔCAN1 Mutation Rate_ratio, which equals the log_2_(Can^R^ rate_yfg__Δ_/Can^R^ rate_wild-type_); (2) how the rate of mutation in the *yfg*Δ *ung1*Δ codeletion strains compared with the mutation rate in the *ung1*Δ strains, Δung1epistasis_ratio, which equals the (log_2_(Can^R^ rate_yfg__Δ *ung1*Δ_/Can^R^ rate_ung1__Δ_); (3) mutational strand bias, ΔStrand Bias_odds-ratio, which equals the log_2_(substitutions at C/substitutions at G nucleotides); and (4) polymerase usage, ΔSubPattern_odds-ratio, which equals the log_2_(C-to-T substitutions/C-to-G substitutions). Nodes of genes associated with sister chromatin cohesion (SCC), DNA replication and recombination, and recombination were identified (black boxes).

We next used unsupervised hierarchical clustering of gene deletion strains based upon changes in strand bias, substitution pattern, epistatic relationship with *ung1*Δ, and Can^R^ rate to see if these features stemmed from general disruption of the common pathways or specific functions of individual genes. As expected, distinct nodes were observed based on this clustering, with one containing all members of the CTF18–RCF complex (*ctf18*Δ, *ctf8*Δ, or *dcc*1Δ), another being associated with replication fork stability factors (*mcm16*Δ, *dia2*Δ, and *tof1*Δ), and a third containing a large number of factors involved in HR (*top3a*Δ, *rad55*Δ, *rad54*Δ, *rad51*Δ, *shu1*Δ, *shu2*Δ, and *csm2*Δ) (Fig. 5; data from Supplemental Table S6). This indicates that the mutagenic features of the genes in these nodes (e.g., leading strand bias for CTF18–RFC factors, epistasis of recombination factors with *ung1*Δ, and increased Rev1 utilization in recombination deficient strains) are generalizable to the entire pathway (Supplemental Fig. S2). However, some notable deviations included clustering of *mph1*Δ with replication fork stability genes and *mrc1*Δ clustering in an undefined node, which indicates that genes with multiple functions may confound grouping via these metrics.

Although A3B was thought to be primarily responsible for APOBEC-induced mutagenesis for most tumor types (Burns et al. 2013a,b), recent studies indicate A3A is the predominate source of APOBEC-induced mutation in breast cancer tumors and potentially in other tumor types (Chan et al. 2015; Cortez et al. 2019; Petljak et al. 2022). To determine if the genes we identified similarly reduce A3A-dependent mutations, we expressed A3A in a subset of yeast deletion strains representing the processes that most significantly reduce A3B-induced mutagenesis. We found that deletion of *HST3* and members of the CTF18–RFC complex (*CTF8*, *CTF18*, and *DCC1*) resulted in the largest increases in A3A-induced mutagenesis (Supplemental Table S2). Strains with gene deletions resulting in defective HR-dependent lesion bypass (*rad54*Δ, *chl1*Δ, *mph1*Δ, *shu1*Δ, and *dia2*Δ) display A3A-dependent mutation rates similar to *ung1*Δ yeast (Supplemental Table S2). These results indicate that the identified genes protect against A3B- and A3A-induced mutagenesis within our yeast model and may function similarly in human tumors with mutagenesis driven by A3A and/or A3B activity.

**Figure 6.**
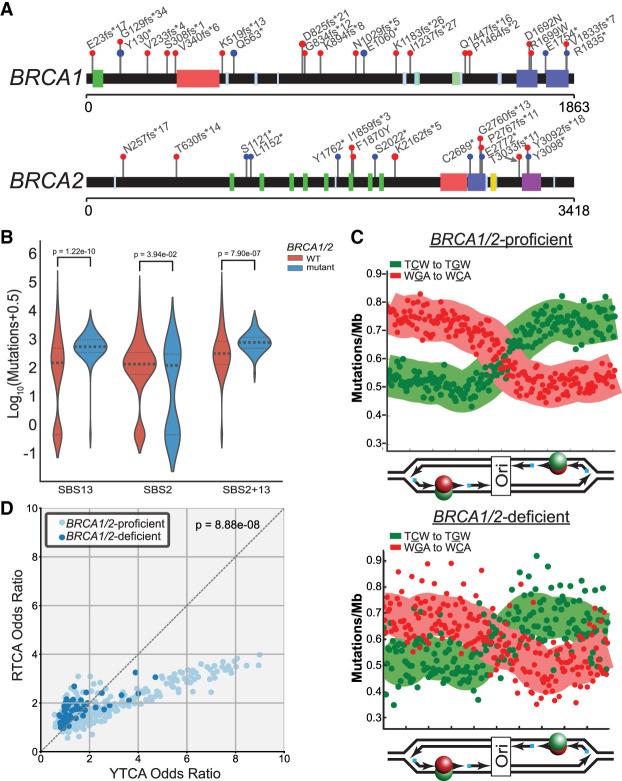
Recombination-deficient breast cancers have elevated APOBEC signature mutagenesis. (*A*) Position of germline mutations in *BRCA1* and *BRCA2* present in 560 breast cancers sequences as part of the International Cancer Genome Consortium. (*B*) Median estimated number of COSMIC SBS2, SBS13, and SBS2 + SBS13 mutations in breast cancers from BRCA-proficient and BRCA-mutant patients. (*C*) Replication strand bias of SBS13 mutations in tumors from BRCA-proficient and BRCA-deficient patients. (*D*) Enrichment of A3B-like (RTCA) and A3A-like (YTCA) signature mutations in BRCA-proficient and BRCA-deficient tumors. Increased representation of the A3B-like mutation signature compared with the A3A-like mutation signature among *BRCA1/2*-mutant tumors was assessed by chi-square test (*P* = 8.88 × 10^−8^).

Upon finding that many proteins involved in HR limit A3B- and A3A-induced mutation in yeast, we assessed if orthologous factors in human tumors have a similar role. We obtained somatic mutations from 560 whole-genome-sequenced breast cancers and assessed the relative abundance of SBS2 and SBS13 in each tumor. Among these tumors, 62 had germline mutations in either *BRCA1* or *BRCA2*, which are required for HR-repair activities in human cells (Fig. 6A). Twenty-nine of 31 *BRCA1* and 30 of 31 *BRCA2* germline mutations were either frameshifts, splice site changes, or nonsense mutations, which generate truncated proteins that likely have reduced, or lost, function(s). We stratified these tumors into either BRCA1/2-proficient or BRCA1/2-deficient classes and determined the median abundance of total APOBEC signature mutations (i.e., SBS2 and SBS13 combined), SBS2 mutations, and SBS13 mutations alone in each class. We found that that BRCA1/2-deficient tumors contained a 2.4-fold higher median load of total APOBEC-induced mutations (*P* < 0.0001 by Mann–Whitney *U* test) compared with BRCA1/2-proficient tumors (Fig. 6B). This increase is largely owing to a 3.8-fold elevation in SBS13 mutations (*P* < 0.0001 by Mann–Whitney *U* test), whereas SBS2 mutations occurred at similar levels between BRCA1/2-proficient and BRCA1/2-deficient tumors (median values of 151 and 142 SBS2 mutations per tumor), respectively. Similar results were observed when BRCA1- or BRCA2-deficient tumors were individually compared with BRCA1/2-proficient tumors, when the presence and absence of SBS3 (a mutation signature indicating HR-deficiency) was used to stratify tumors, or when HR deficiency was likely to have been acquired somatically as opposed to in the germline (Supplemental Figs. S3, S4). The increase of only SBS13 mutations in BRCA1/2-deficient breast cancers mirrors our observation that increased APOBEC-induced mutation is driven by higher rates of C-to-G substitutions in recombination-deficient yeast strains (Fig. 4B). Therefore, these results suggest that BRCA1 and BRCA2 likely limit APOBEC signature mutations in human cancer cells by participating in HR-mediated lesion bypass of base lesions in the lagging strand template. To determine if BRCA1/2 may facilitate HR-dependent lesion bypass of APOBEC-induced DNA damage in breast cancer, we assessed SBS13 mutations for replicative asymmetry associated with deamination of the lagging strand template in the absence of BRCA1 or BRCA2 (Fig. 6C). As previously reported for pan-cancer analysis of APOBEC-induced mutations (Haradhvala et al. 2016; Morganella et al. 2016; Seplyarskiy et al. 2016), SBS13 mutations in BRCA1/2-proficient breast cancers associated strongly with deamination of cytidines in the lagging strand template. SBS13 mutations in BRCA1/2-deficient tumors displayed the same replicative asymmetry, indicating the primary source of APOBEC-induced mutation was owing to defective HR-dependent lesion bypass or possibly lagging strand–specific ssDNA gaps found in BRCA1/2-deficient tumors (Cong et al. 2021; Panzarino et al. 2021) as opposed to ssDNA formed at failed DSB repair intermediates. Furthermore, we assessed whether BRCA1/2-deficient tumors contain an overrepresentation of C-to-G and C-to-T substitutions in either the YTCA or RTCA motifs, which correspond to A3A-like or A3B-like mutation signatures, respectively (Chan et al. 2015). This analysis indicated that BRCA1/2-deficient tumors were more likely to be enriched for A3B-like signature mutations (Fig. 6D), possibly owing to lethality between BRCA1/2 deficiency and the higher amounts of damage caused by A3A.

**Figure 7.**
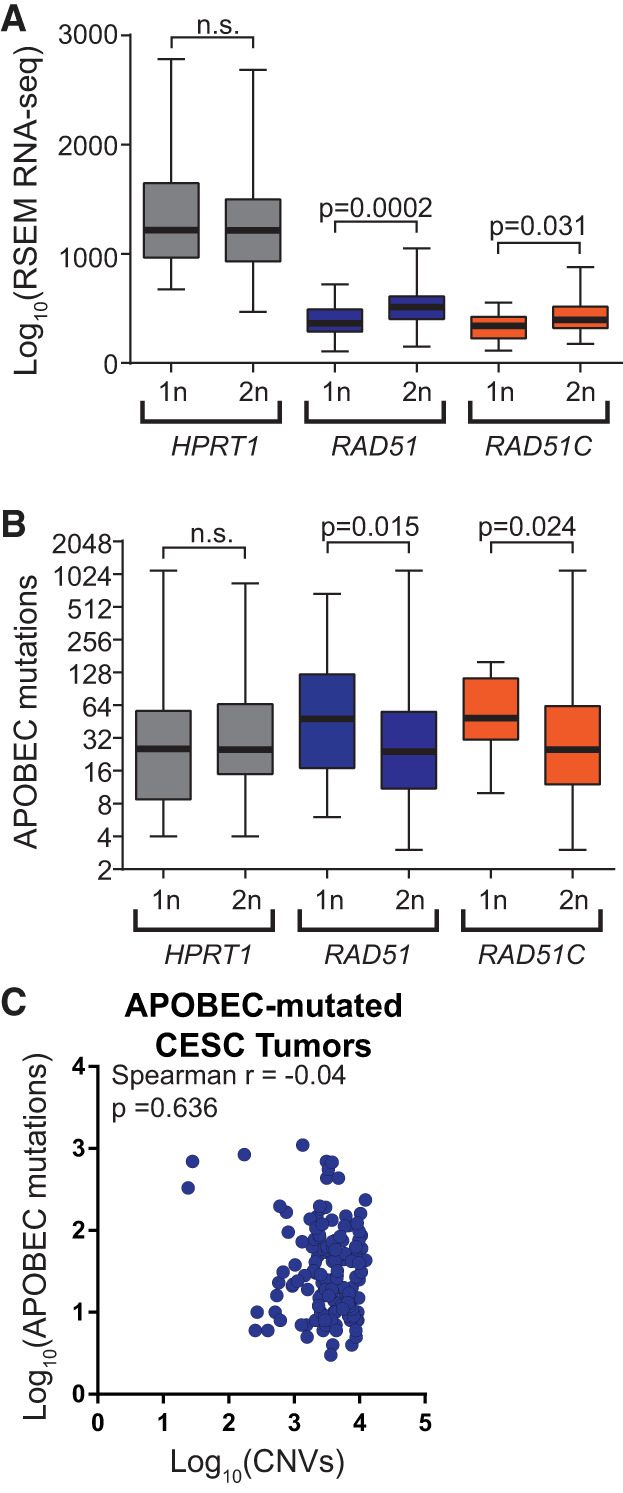
Impact of recombination gene haplo-insufficiency on APOBEC-induced mutagenesis in cervical cancers. (*A*) Median RSEM normalized RNA-seq expression values (*middle* horizontal bar in each box plot) for *HPRT1*, *RAD51*, and *RAD51C* in cervical cancers; one or two copies of each gene as determined by GISTIC analysis of SNP6 array data. Error bars indicate the range of RSEM expression values among tumors in each group. Tumors with two copies of *RAD51* and *RAD51C* have higher expression of the respective genes than tumors with one copy as assessed by Mann–Whitney ranked sum test. (*B*) Minimal estimate of the number of APOBEC-induced mutations in cervical cancers with one or two copies of *HPRT1*, *RAD51*, and *RAD51C*. Median values are indicated by the middle bar in the box plots, and error bars indicate the range of values in each group. (*C*) Lack of correlation between the number of copy number variants (CNVs) and APOBEC-induced mutations in cervical cancers sequenced by TCGA. The Spearman's coefficient (rho) and *P*-value are indicated.

Next, we determined if additional HR factors protect against APOBEC signature mutations like *BRCA1/2* by using somatic base substitutions, copy number variations, and RNA-seq expression data from 309 cervical squamous cell carcinoma and endocervical adenocarcinoma (CESC) tumors characterized by The Cancer Genome Atlas (TCGA). Among CESC tumors, we identified 148 samples with a statistical overrepresentation of APOBEC signature mutations. By GISTIC analysis, a single copy of either *RAD51*, *RAD51C*, or *HPRT1* was lost in 31, 12, and 30 of the APOBEC-mutated CESC tumors, respectively. Tumors containing a single *RAD51* or *RAD51C* allele displayed 1.42-fold and 1.16-fold lower median gene expression than copy-neutral tumors (*P* = 0.0002 and *P* = 0.0314, respectively, by Mann–Whitney *U* test) (Fig. 7A), indicating that the loss of one allele of these genes corresponds to a lower transcript level and likely decreases protein abundance. Consequently, a single-allele deletion of either *RAD51* or *RAD51C*, but not the housekeeping gene *HPRT1,* correlated with higher minimal estimates of total APOBEC-induced mutations (*P* = 0.015 and *P* = 0.0243, respectively, by Mann–Whitney *U* test) (Fig. 7B). Importantly, within this CESC data set, unlike data sets from most APOBEC-mutated tumor types, the overall number of copy number changes within tumors does not correlate with the number of base substitutions (Fig. 7C), ensuring that the associations we observed between somatic single-gene deletion in *RAD51* and *RAD51C* are unlikely to result from tumors with higher copy number burden and thus are more likely to have deletions in *RAD51* or *RAD51C*, having de facto higher mutation burdens. Thus, single-allele deletions of *RAD51* and *RAD51C* appear to be haplo-insufficient in limiting APOBEC-induced mutation, which further supports a model in which HR factors are used to bypass APOBEC-induced abasic sites to reduce APOBEC-signature mutations in multiple human cancer types.

## Discussion

Using a yeast deletion screen, we identified 61 strains with gene deletions that significantly increased A3B-induced mutagenesis (Fig. 1B). Genes encoding proteins functioning in DNA replication, HR, replication, and chromatin modification were enriched among genes that protect against APOBEC-generated mutations (Fig. 2). By measuring the A3B-induced mutation rates in yeast strains containing each gene deletion combined with *ung1*Δ and generating *CAN1* mutation spectra from A3B-expressing strains containing single-gene deletions, we were able to generate discrete characteristics associated with defective processes that increase APOBEC3B-induced mutagenesis (summarized in Fig. 5).

We previously found that Mph1 reduces A3B-induced mutagenesis during DNA replication via its role in HR-dependent lesion bypass downstream from *ung1*Δ (Hoopes et al. 2017). We also found that the Shu-complex members *CSM2*, *SHU1*, and *SHU2* are important for HR-dependent lesion bypass of APOBEC-induced abasic sites (Rosenbaum et al. 2019). In this study, we found that many gene deletions in yeast with primary and supporting roles in HR (i.e., *top3a*Δ, *rad55*Δ, *rad54*Δ, *rad51*Δ, *sgs1*Δ, *mph1*Δ, *mec3*Δ, and *ddc1*Δ) also increased APOBEC-induced mutagenesis to levels like those of *ung1*Δ yeast. Deletion of almost all genes with roles in HR produced similar A3B-induced *CAN1* mutation rates when deleted in isolation or in combination with *ung1*Δ, which indicates they likely function downstream from Ung1 in prevention of APOBEC-induced mutations. Because loss of HR-dependent bypass results in a mutation rate similar to that of the loss of Ung1, we believe HR-dependent bypass is the only pathway in yeast that prevents abasic sites generated as a consequence of APOBEC activity during DNA replication from becoming mutations. Also, deletion of genes with roles in HR significantly increased the C-to-G to C-to-T ratio among sequenced *CAN1* mutants, which indicates greater usage of Rev1-mediated TLS bypass of APOBEC-dependent abasic sites and indicates the encoded proteins reduce APOBEC-induced mutations by participating in HR-dependent lesion bypass (data summarized in Fig. 5). Consequently, the loss of Ung1 and HR-mediated bypass are not truly epistatic. Instead, loss of each process channels APOBEC-generated dU and abasic sites into separate pathways that always result in mutation, but with different mutagenic outcomes. Similarly, loss of Rev1 catalytic activity in strains deficient in HR-mediated lesion bypass results in no change in the mutation rate but alters the mutation spectra as abasic sites are channeled into bypass via an A-rule polymerase (i.e., Pol-delta and/or Pol-zeta). A summary of the roles of Ung1, HR-dependent lesion bypass, and TLS can be found in Supplemental Fig. S1A. Although we have not found alternate mechanisms that prevent APOBEC-induced mutagenesis, we reason they may exist for cells showing replication or transcriptional stress that could, in theory, shift the predominate available target of APOBECs from the lagging strand template during replication to ssDNA to a different context. For example, BER has been shown to limit APOBEC3B-induced mutation in highly transcribed tRNA genes (Saini et al. 2017).

We found that BRCA1/2-deficient tumors have an elevated abundance of APOBEC-induced mutations owing primarily to increased SBS13 mutations, which arise from REV1-dependent TLS (Petljak et al. 2022). These APOBEC-signature mutations in BRCA1/2-deficient tumors have replication symmetry indicative of mutations arising from the activity of APOBECs on the lagging strand template during DNA replication. Although several studies indicate HR-dependent lesion bypass occurs in human cells (Adar et al. 2009; Taglialatela et al. 2021), this process remains poorly understood. Our results suggest that BRCA1/2 function in the HR-dependent bypass of lesions that block DNA replication and that this repair pathway is used to reduce APOBEC-induced mutations in tumor cells, helping to define some of the genetic components of this pathway.

The deletion of *HST3* resulted in the largest increase in the rate of A3B-induced *CAN1* mutations. Hst3 is a H3 histone deacetylase (HDAC) and member of the sirtuin family of deacetylases (Brachmann et al. 1995). Newly synthesized histones are acetylated by the actions of a histone chaperone, Asf1, and the histone acetyltransferase Rtt109 (Driscoll et al. 2007; Tsubota et al. 2007) to mark new histones that are incorporated into DNA behind the replication fork with H3K56ac. Hst3 functions to deacetylate H3K56 during G_2_/M phases of the cell cycle. The *hst3*Δ, *asf1*Δ, and *rtt109*Δ yeast strains elevated the rate A3B-induced mutagenesis at *CAN1* by 10.0-, 1.96-, and 1.77-fold, respectively, which indicates both establishment and removal of H3K56ac are important for preventing APOBEC-induced mutagenesis. Epistasis between *asf1*Δ or *rtt109*Δ and *ung1*Δ for A3B-induced mutagenesis likely results from the importance of postreplicative establishment of H3K56ac for homology-directed repair. The A3B-induced mutation rate in *hst3*Δ stains is 1.8-fold higher than that of *ung1*Δ and similarly higher than rates for strains with defective HR-dependent lesion bypass, which indicates *hst3*Δ likely increases the amount of ssDNA that can be deaminated by APOBECs. However, the mutation rate in *hst3*Δ *ung1*Δ yeast is not higher than that of *hst3*Δ alone. Loss of Hst3 could result in increased ssDNA in the context of DNA replication and loss of HR-dependent lesion bypass. Our data showing *hst3*Δ has increased lagging strand bias similar to strains with defective HR-mediated bypass and previous studies indicating the establishment and removal of H3K56ac during and after replication are important for repair via HR (Thaminy et al. 2007; Muñoz-Galván et al. 2013) suggest that this explanation is most likely (see model in Supplemental Fig. S1B). Combining *hst3*Δ with *asf1*Δ or *rtt109*Δ suppresses A3B mutagenesis to levels observed in *asf1*Δ or *rtt109*Δ strains (Supplemental Table S2), which suggests persistent H3K56ac is responsible for elevated A3B-induced mutagenesis in *hst3*Δ strains. This result is similar to those for spontaneous increases in the *CAN1* mutations rate for *hst3*Δ, *hst4*Δ strains, which are repressed by loss of *ASF1* or *RTT109* (Kadyrova et al. 2013). Because defective H3K56ac deacetylation has been implicated in control of origin firing, DNA replication efficiency, limiting break-induced replication and mutations arising from replication, DNA damage response signaling, cohesion establishment, and HR (Thaminy et al. 2007; Celic et al. 2008; Muñoz-Galván et al. 2013; Che et al. 2015; Irene et al. 2016; Simoneau et al. 2016; Gershon and Kupiec 2021), further work is required to parse out the mechanism(s) by which H3K56ac influences APOBEC-induced mutation. Two additional deletions of genes encoding HDACs, *hos1*Δ and *hst1*Δ, also increased A3B-induced mutagenesis but were dissimilar to *hst3*Δ for characteristics of A3B-induced mutagenesis (Fig. 5). Deletions of other HDACs, *hda1*Δ, *hda2*Δ, *hos2*Δ, *hos3*Δ, *hst2*Δ, *hst4*Δ, and *sir2*Δ, did not significantly increase A3B-induced mutagenesis (Supplemental Table S2). In human cells, SIRT1, SIRT2, SIRT6, and HDAC1/2 have all been implicated in H3K56ac deacetylation in different contexts (Das et al. 2009; Michishita et al. 2009; Yuan et al. 2009; Zhu et al. 2015). Reports of elevated H3K56ac in human tumor samples (Das et al. 2009), the potential of activated RAS-PI3K signaling to reduce H3K56ac (Liu et al. 2012), and a large number of clinical trials for HDAC inhibitors that likely increase H3K56ac levels in tumor cells (Eckschlager et al. 2017) all suggest changes in H3K56ac levels may be widespread in cancer. These cancer-specific modulators of H3K56ac may affect APOBEC-induced mutagenesis in tumors, which could influence patient prognosis and influence therapeutic outcomes.

Deletion of *CTF18*, *CTF8*, and *DCC1* caused some of the largest increases of APOBEC-induced mutagenesis we observed. The encoded proteins Ctf18, Cft8, and Dcc1 coimmunoprecipitate with Rfc2, Rfc3, Rfc4, and Rfc5 (Mayer et al. 2001) to form the CTF18–RFC complex, which is known to function in multiple processes. APOBEC-induced *CAN1* mutations for *ctf8*Δ, *ctf18*Δ, and *dcc1*Δ were biased to the leading strand, and when these deletions were combined with *ung1*Δ, they led to a greater than additive increase in the A3B-induced *CAN1* mutation rate, which indicates these deletions elevate APOBEC-generated mutagenesis by increasing the availability of ssDNA on the leading strand during DNA replication (summarized in Fig. 5; Supplemental Fig. S1C). Although CTF18–RFC has well-characterized roles in formation of chromatid cohesion (Mayer et al. 2001), characteristics of A3B-induced mutations in *ctf18*Δ, *ctf8*Δ, and *dcc1*Δ strains are different from other deleted genes with roles in cohesion (i.e., *sgs1*Δ, *top3*Δ, *chl1*Δ, and *mrc1*Δ). CTF18–RFC has been implicated in playing a role during HR (Ogiwara et al. 2007); however, defective HR-dependent lesion bypass tends to be epistatic with *ung1*Δ and increases APOBEC mutagenesis primarily on the lagging strand, which is contrary to the effect of defective CTF18–RFC upon APOBEC mutagenesis. Furthermore, CTF18–RFC participates in Mrc1-dependent activation Rad52 within the DNA replication checkpoint (Garcia-Rodríguez et al. 2015). However, defects in CTF18–RCF and *mrc1*Δ have opposing effects on both the strand bias and use of Rev1-mediated TLS in respect to APOBEC-induced mutations for *CAN1*, which indicates they affect APOBEC-induced mutagenesis in distinct ways. Defects in the CTF18–RFC complex also make yeast sensitive to hydroxyurea and MMS and cause gross chromosomal rearrangements, phenotypes apparently linked to defective PCNA loading (Okimoto et al. 2016). CTF18–RFC colocalizes with the replication fork upon HU treatment and during unperturbed replication (Liu et al. 2020) and directly interacts with polymerase epsilon (Garcia-Rodríguez et al. 2015; Okimoto et al. 2016), which together indicate CTF18–RFC is important for the efficiency of replication and/or facilitating bypass DNA damage on the leading strand. Our results indicate that a defective CTF18–RFC complex increases ssDNA available for APOBEC activity on the leading DNA strand, which further supports a role for PCNA loading by CTF18–RFC on the leading strand in preventing or repairing DNA damage.

We identified many genes without clear functional roles that might protect against A3B-mediated mutagenesis. Among those with the highest APOBEC-induced mutagenesis include *trm10*Δ, *dia2*Δ, *yaf9*Δ, and *ypl077c*Δ. *TRM10* encodes a tRNA methyltransferase, and there is no obvious explanation for it protecting against A3B-induced mutagenesis. TRM10 is elevated upon induction of replication stress (Tkach et al. 2012), indicating it may be needed for recovery of these conditions. Also, *TRM10* is adjacent to *RFC4*, and *trm10*Δ might increase replication stress by reducing *RFC4* expression. Deletions of *DIA2*, *YAF9*, and *YPL0177*c all increase lagging strand bias and Rev1 usage for A3B-induced mutations, which is consistent with the encoded proteins participating in HR-mediated bypass. The Yaf9-containing NuA4 HDAC complex and Dia2 have been implicated in regulating HR (Bennett and Peterson 2015; Chaudhury and Koepp 2017). However, when *DIA2*, *YAF9*, and *YPL0177*c deletions are combined with *ung1*Δ, they cause higher mutation rates than *ung1*Δ alone, which indicates these defects may also increase ssDNA. Consequently, these genes may protect against APOBEC-induced mutagenesis via multiple functions and should be investigated for additional roles in DNA repair and replication.

Although our screening efforts identified many deletions that increased APOBEC-induced mutagenesis, the list of deletions we found is likely not comprehensive. Significant variability exists in individual mutation frequency measurements, which likely resulted in missed hits during the first round of our screening process. In fact, some of the candidates that were characterized to better identify processes affecting A3B-induced mutagenesis that have elevated A3B-induced *CAN1* mutation rates and altered mutation spectra, were missed in the original screening process. Our screen was also limited to nonessential gene deletions. It is likely that tumor cells could also have elevated APOBEC-induced mutagenesis owing to mutations, reduced expression, or altered post-translational modifications that reduce the function of essential genes like RPA and replicative DNA polymerases (Sui et al. 2020). Also, we anticipate that additional genes not represented in the yeast genome likely limit APOBEC-induced mutations in human cells.

Our finding that defective HR in breast and cervical cancer tumors increases APOBEC-induced mutagenesis is a good indicator that the genes we identified in yeast as restrictors of APOBEC mutagenesis may do so in the context of human tumors. Because APOBEC-induced dUs are converted into strand-specific abasic sites in the presence of uracil DNA glycosylase, the spectra and distribution of APOBEC-induced mutations can be used to define roles of DNA repair and replication proteins in abasic site bypass and to identify proteins that protect against aberrant ssDNA formation. For these reasons, we believe that in addition to identifying and characterizing novel modulators of APOBEC-induced mutagenesis, these findings will facilitate efforts to study many processes in yeast and human cells.

## Methods

### Generating a library of haploid yeast deletion strains with A3B expression

Yeast strain yTM-02 was transformed with a *LEU2*-marked ARS/CEN plasmid expressing APOBEC3B, pTM-021 (Rosenbaum et al. 2019). yTM-02 + pTM21 was mated to each BY4741 deletion strain, and diploids were selected on SC-leu + G418 media. The resulting diploid strains were replica-plated to sporulation media. After 10 d, sporulation plates were replica-plated to SC-his-leu + G418 + cycloheximide to select for haploid cells with individual gene deletions and expressing A3B. Clonal isolates of each deletion strain expressing A3B were generated by streaking to single colonies on SC-his-leu + G418. Strain genotypes can be found in Supplemental Table S7, and the primer sequences used to construct and verify gene deletions are in Supplemental Table S8.

### Measurement of mutation frequencies and rates

To determine yeast Can^R^ frequencies during the screening process, colonies grown to approximately 1 × 10^7^ cells were resuspended in H_2_O, and about 200 yeast cells were plated for nonselective media and 2.5 × 10^5^ cells for A3B-expressing strains, or 1 × 10^7^ cells for strains with the empty vector control were plated on SC-arg + canavanine media. *CAN1* mutation rates (i.e., mutations per generation) for all figures and in Supplemental Table S1 were calculated from eight or more mutation frequency measurements by the maximum likelihood method (Zheng 2002) using either FALCOR (Hall et al. 2009) or the fluxer.R script (https://github.com/barricklab/barricklab/blob/master/fluxxer.R) run in R (R Core Team 2022).

### Screen for genetic defects that increase APOBEC-induced mutations

The screening process for gene deletions that increase A3B-induced mutations was conducted in three rounds. In round one, a single mutation frequency was measured for each deletion strain expressing A3B. For deletion strains from round one with a Can^R^ frequency greater than or equal to twofold higher than the median mutation frequency, a second Can^R^ frequency was measured. For the 273 strains from round two with a Can^R^ frequency twofold or more higher than the median mutation frequency, new clonal isolates from the population of haploid cells were generated and colonies for Can^R^ frequencies were all grown to 4 × 10^6^ cells, and four additional Can^R^ frequencies were measured. Deletion strains with a median mutation frequency twofold or more higher than that of wild-type BY4741 were labeled as hits from the screening process. The gene deletion in each of the 101 screen-derived candidates was verified by sequencing the unique barcode associated with deletion.

### Post screen characterization of screen hits and candidates

For the 101 screen-identified candidates and an additional 76 candidates that were chosen from GO analysis of the screen-derived candidates, we obtained the original deletion strain from the BY4741 library or made the strain de novo from the parental BY4741 strain. These strains were transformed with either pySR-419, an empty vector control plasmid, or pSR-440, a hygromycinB resistance–marked ARS/CEN plasmid expressing APOBEC3B (Hoopes et al. 2016) and used for mutation rate measurements and generation on mutation spectra.

### Determination of *CAN1* mutation spectra

Independent clonal yeast colonies were grown to about 1 × 10^7^ cells and replica-plated to SC-arg + canavanine media. Can^R^ papillae were subjected to an additional round of clonal expansion via single colony streaking. Genomic DNA was extracted from the clonal Can^R^ mutants using previously described techniques and used as templates for PCR amplification of *CAN1* using primer pairs with unique combinations of barcodes, (Supplemental Table S8). Approximately 2500 amplicons were subjected to single-molecule, real-time (SMRT) sequencing on either a Pacific Biosciences (PacBio) RSII or PacBio sequel instruments.

### Statistical comparisons of A3B-induced mutation in yeast gene deletion strains

Gene deletion strains with elevated A3B-induced mutation were statistically identified as those with 95% confidence intervals that were nonoverlapping compared with wild-type yeast expressing A3B for the median Can^R^ rate determined by the maximum likelihood method in FALCOR. The resulting 61 genes were clustered based on related function using STRINGdb (Szklarczyk et al. 2019; www.sting-db.org) set to the highest-confidence linkage and with *k*-means clustering of eight nodes. Enriched component and process GO categories among these genes were also determined with STRINGdb. Full lists of enriched categories are provided in Supplemental Tables S9 and S10. Whether specific gene deletions were epistatic, additive, or above-additive with *ung1*Δ in regard to the A3B-induced Can^R^ rate was determined by adding the appropriate bounds of the 95% confidence intervals for the single-deletion strains to the *ung1*Δ (i.e., upper bound to upper bound and lower bound to lower bound) and comparing these combined bounds to the experimentally determined confidence intervals of the double-deletion strains and the *ung1*Δ strain. Epistatic genes contained overlapping experimentally measured confidence intervals with the single *ung1*Δ strain. Additive genes had overlapping measured confidence intervals with those adding the corresponding single-deletion strains’ bounds together. Above-additive genes had higher experimentally determined rates with nonoverlapping 95% confidence intervals compared with the estimate calculated by adding bounds from the single-deletion strain to the single *ung1*Δ strain. Altered strand biases were identified by the pairwise comparison of the number of C and G substitutions in sequenced *can1* alleles from a specific gene deletion strain expressing A3B to those from wild-type yeast expressing A3B by two-sided Fisher's exact test. Altered Rev1-mediated bypass was determined by comparing the ratio of C-to-T and C-to-G substitutions in *can1* from individual gene deletion stains expressing A3B compared pairwise with the same ratio from A3B-expressing wild-type yeast by two-sided Fisher's exact test. All *P*-values were corrected for multiple hypothesis testing by the Bonferroni method (Bland and Altman 1995). Hierarchical clustering of gene deletion phenotypes was conducted through the Morpheus webtool (https://software.broadinstitute.org/morpheus/), using average linkage of the one minus Pearson's correlation of log_2_ adjusted values.

### Primary human tumor analyses

Presence of germline mutations in the *BRCA1* and *BRCA2* genes were listed in Supplemental Table 20 of the work of Nik-Zainal et al. (2016). Abundances of signature 2 and signature 13 mutations per tumor were obtained from Supplemental Table 21 of the work of Nik-Zainal et al. (2016). Putative APOBEC signature C-to-T and C-to-G mutations were identified among the total list of somatic mutations for whole-genome-sequenced breast cancers (ICGC data set) and exome-sequenced cervical cancers (TCGA data set) using the APOBECng module integrated within the firehose pipeline (Roberts et al. 2013). Comparisons of APOBEC-induced mutation load between BRCA1/2-proficient and BRCA1/2-deficient tumors, as well as between minus one and copy-neutral *HPRT1*, *RAD51*, and *RAD51C* cervical cancers, were conducted by a Mann–Whitney rank sum test. Somatic C > K mutations in YTCA, RTCA, and NNCN contexts were counted from individual BRCA1/2-proficient and BRCA1/2-deficient tumor data. These counts along with the occurrence of corresponding tetranucleotides within the hg19 genome (selected over the newer GRCh38 reference to provide a more accurate determination of mutation frequency as hg19 was used for alignment of the published tumor sequencing data) were used to calculate the A3B-like (RTCA) and A3A-like (YTCA) substitution odds ratios similar to previous methods (Chan et al. 2015). The bias toward the A3B mutation signature in BRCA1/2-deficient tumors was evaluated by a Fisher's exact test. TCW-to-TGW mutations from BRCA1/2-proficient and BRCA1/2-deficient tumors with a statistical enrichment of APOBEC signature mutations (determined by Fisher's exact test and Benjamini–Hochberg [Benjamini and Hochberg 1995] multiple testing correction as previous work) (Roberts et al. 2013) were assessed for transcriptional and replicative asymmetry using the asymmetry module in MATLAB (Haradhvala et al. 2016).

### Additional methods

Detailed methods for the creation of yeast strains and plasmids, RT-qPCR, and a full description of data sources can be found in the Supplemental Materials.

## Data access

All raw PacBio sequencing data generated in this study have been submitted to the NCBI BioProject database (https://www.ncbi.nlm.nih.gov/bioproject/) under accession number PRJNA977835.

## Supplementary Material

Supplement 1

Supplement 2

Supplement 3

Supplement 4

Supplement 5

Supplement 6

Supplement 7

Supplement 8

Supplement 9

Supplement 10

Supplement 11
