# Supplementary material for: Genetic inhibitors of APOBEC3B-induced mutagenesis
Source: Genome Res. 2023 Sep;33(9):1568–81. doi: 10.1101/gr.277430.122 (PMC10620048; doi:10.1101/gr.277430.122)
Supplement: Supplement 11 [file Supplemental_Information_Final.pdf]

SUPPLEMENTAL INFORMATION

SUPPLEMENTAL METHODS

Yeast growth conditions and strains

Yeast strains were grown on YPDA or synthetic complete (SC) dropout media (Burke et al. 2000). The strains used in this study are all derived from BY4742 or BY4741 and the library of BY4741 deletion strains was purchased from Open Biosystems (currently available from Transomic Technologies; Huntsville, AL). The genotype of strain HMK246, which was created by the Lobachev lab (Zhang et al. 2012) from BY4742, is: MATα, ura3-Δ, leu2-Δ, his3-Δ, lys2-Δ, rpl28-Q38K, mfa1Δ::MFA1pr-HIS3, V34205::lys2::(GAA)230, V29617::hphMX. Strain HMK246 was further modified by exchanging the hphMX selection cassette for natMX to create strain yTM-01. Strain yTM-01 was modified by converting the V34205::lys2::(GAA)230 allele to a functional *LYS2* allele to generate strain yTM-02. Strain yTM-03 was created by using a PCR-generated cassette to delete *UNG1* in the yTM-02 strain.

UNG1 deletion strains were created by either transforming yeast strains with PCR-generated NatMX deletion cassettes or by a mating yTM-03 and strains from the deletion library, sporulating, and selecting haploid yeast on SC-Leu+G418. Yeast strains with the *rev1-D467A, E468A* mutation were generated with CRISPR-Cas9 using methods as in (Vandenberg et al. 2023) and homology directed repair donor oligos SAR616 and SAR617. The *rev1-D467A, E468A* strains were validated by PCR amplification using oligos oTM-1200 and oTM-1203 and Sanger sequencing with oligo SAR622. For genotypes and additional details about individual strains, (see Supplemental Table S7) and for primer sequences used to construct and verify gene deletions (see Supplemental Table S8).

Plasmid construction

The A3A yeast expression plasmid, pVH-A3A, was constructed by ligating a DraI and PfimI (NEB) digested gBlock containing the A3A CDS with the yeast ACT1 intron inserted 20 nucleotides 3’ of the A3A start codon (i.e. SRO235; gBlocks® Gene Fragment technology; IDT)

into the PmeI and PflmI restriction sites of a previously constructed yeast A3A expression vector, pSR435 (Chan et al. 2015). Correct cloning was confirmed by Sanger sequencing. Sequencing of RT-PCR products for the exogenously expressed A3A from pVH-A3A in yeast with primer SRO251(Supplemental Table S8) confirmed accurate splicing of the A3A mRNA.

RT-qPCR to assess changes in A3B expression.

Yeast strains containing pSR-440 (A3B expression) were grown overnight in 20 mL of YPDA+hygromycinB and cell pellets from these cultures were frozen in liquid nitrogen. RNA was extracted from these pellets using TRIzol and glass beads, which was followed by a second purification using the E.Z.N.A. Total RNA Kit I (Omega Biotek) with an on-column DNase I digest. cDNA was generated using MashUp RT, a reverse transcriptase based on FeLV-RT, which was purified as in (Alekseenko et al. 2021). qPCR was performed using Forget-Me-Not EvaGreen qPCR Master Mix (Biotium) and primers oTM-115 and oTM-117 (A3B specific) and oTM-1348 and oTM-1349 (*ALD6* specific) (Supplemental Table S8).

Description of data sources for bioinformatics analysis

Somatic mutations from whole genome sequenced breast cancers from the International Genome Consortium were obtained from https://dcc.icgc.org/api/v1/download?fn=/release_26/Projects/BRCA-EU/simple_somatic_mutation.open.BRCA-EU.tsv.gz. Copy number variations, somatic mutation lists and RSEM normalized RNA-seq expression data for CESC tumors were obtained from the TCGA at http://gdac.broadinstitute.org/runs/analyses__2016_01_28/data/CESC-TP/20160128/gdac.broadinstitute.org_CESC-TP.CopyNumber_Gistic2.Level_4.2016012800.0.0.tar.gz, http://gdac.broadinstitute.org/runs/analyses__2016_01_28/data/CESC-TP/20160128/gdac.broadinstitute.org_CESC-TP.Mutation_APOBEC.Level_4.2016012800.0.0.tar.gz, and

http://gdac.broadinstitute.org/runs/stddata__2016_01_28/data/CESC/20160128/gdac.broadinstitute.org_CESC.Merge_rnaseqv2__illuminahiseq_rnaseqv2__unc_edu__Level_3__RSEM_genes_normalized__data.Level_3.2016012800.0.0.tar.gz, respectively.

SUPPLEMENTAL REFERENCES

Alekseenko A, Barrett D, Pareja-Sanchez Y, Howard RJ, Strandback E, Ampah-Korsah H, Rovšnik U, Zuniga-Veliz S, Klenov A, Malloo J et al. 2021. Direct detection of SARS-CoV-2 using non-commercial RT-LAMP reagents on heat-inactivated samples. *Scientific Reports* **11**: 1820.

Burke D, Dawson D, Stearns T. 2000. Methods in Yeast Genetics: A Cold Spring Harbor Laboratory Course Manual (2000 Edition). *Plainview, NY: Cold Spring Harbor Laboratory Press[Google Scholar]*.

Chan K, Roberts SA, Klimczak LJ, Sterling JF, Saini N, Malc EP, Kim J, Kwiatkowski DJ, Fargo DC, Mieczkowski PA et al. 2015. An APOBEC3A hypermutation signature is distinguishable from the signature of background mutagenesis by APOBEC3B in human cancers. *Nature genetics* **47**: 1067-1072.

Vandenberg BN, Laughery MF, Cordero C, Plummer D, Mitchell D, Kreyenhagen J, Albaqshi F, Brown AJ, Mieczkowski PA, Wyrick JJ et al. 2023. Contributions of replicative and translesion DNA polymerases to mutagenic bypass of canonical and atypical UV photoproducts. *Nat Commun* **14**: 2576.

Zhang Y, Shishkin AA, Nishida Y, Marcinkowski-Desmond D, Saini N, Volkov KV, Mirkin SM, Lobachev KS. 2012. Genome-wide screen identifies pathways that govern GAA/TTC repeat fragility and expansions in dividing and nondividing yeast cells. *Mol Cell* **48**: 254-265.

SUPPLEMENTAL FIGURES

Supplement Figure S1

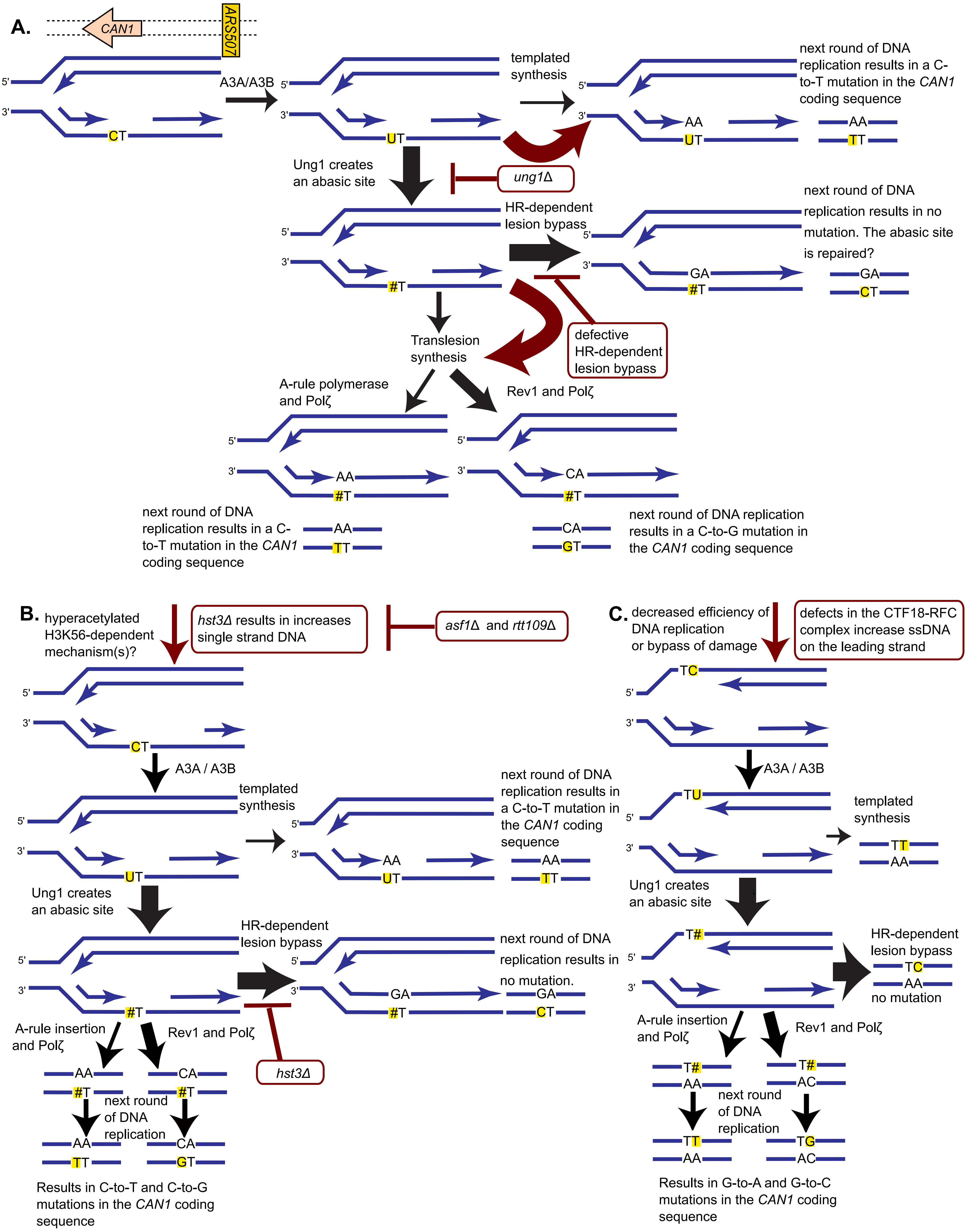

Supplemental Figure S1. Models for increased APOBEC-induced mutagenesis. Data from https://cerevisiae.oridb.org indicates that the *CAN1* gene is replicated most often from *ARS507*, which places dC residues of the *CAN1* coding sequence on the lagging strand template during DNA replication. This observation is consistent with our data showing APOBEC-induced mutations at dC residues are 2.1-fold more frequent than those at dG residues. Blue arrows in the depiction of replication forks indicate DNA synthesis. Black arrows indicate steps that control outcomes of dC residues deaminated by A3B and are weighted more heavily for steps that lead to the predominant outcomes. Maroon arrows indicated how outcomes are changed by defective processes. The dC residues deaminated by A3B are highlighted yellow throughout the figure. (A) Outcomes of APOBEC-induced deamination in wild type yeast and strains lacking Ung1 or HR-dependent lesion bypass. Approximately 80% of APOBEC-generated dU residues are converted to abasic sites by Ung1. In wild type yeast, the vast majority of these abasic sites are channeled into HR-dependent lesion bypass, which avoids mutations that would be generated by TLS. Loss of Ung1 results in all APOBEC-generated dU residues templating during DNA replication resulting in C-to-T mutations. Loss of HR-dependent lesion bypass results in abasic sites being bypassed by TLS with insertion of dC across form the abasic site by Rev1 and extension by Polζ being the predominate mechanism, which results in increased C-to-G mutations. (B) Deletion of *HST3* led to the largest increase in A3B-induced mutagenesis and a mutation rate approximately twice that of *ung1*Δ yeast, which would indicate *hst3*Δ increases ssDNA. This large increase in A3B induced mutagenesis is suppressed by deletion of either *ASF1* or *RTT109*, which indicated it is dependent on hyperacetylated H3K56. Interestingly, combining *ung1*Δ and *hst3*Δ did not further increase APOBEC-induced mutagenesis, which indicates a lack of HR-meditated bypass in *hst3*Δ yeast, which is consistent with literature indicating establishment and removal of H3K56ac is important for multiple aspects of homologous recombination. (C) Defects in the CTF18-RFC complex increase in the mutation rate greater than *ung1*Δ alone and further increase the mutation rate when combined with *ung1Δ*, which indicates that deletion of *CTF8*, *CTF18*, and *DCC1* likely increase ssDNA. A significant bias to dG residues in the *CAN1* coding sequence points to elevated ssDNA on the leading strand, which likely results from decreased loading/unloading of PCNA on the leading strand and reduced efficiency of DNA replication and/or lesion bypass.

Supplemental Figure S2

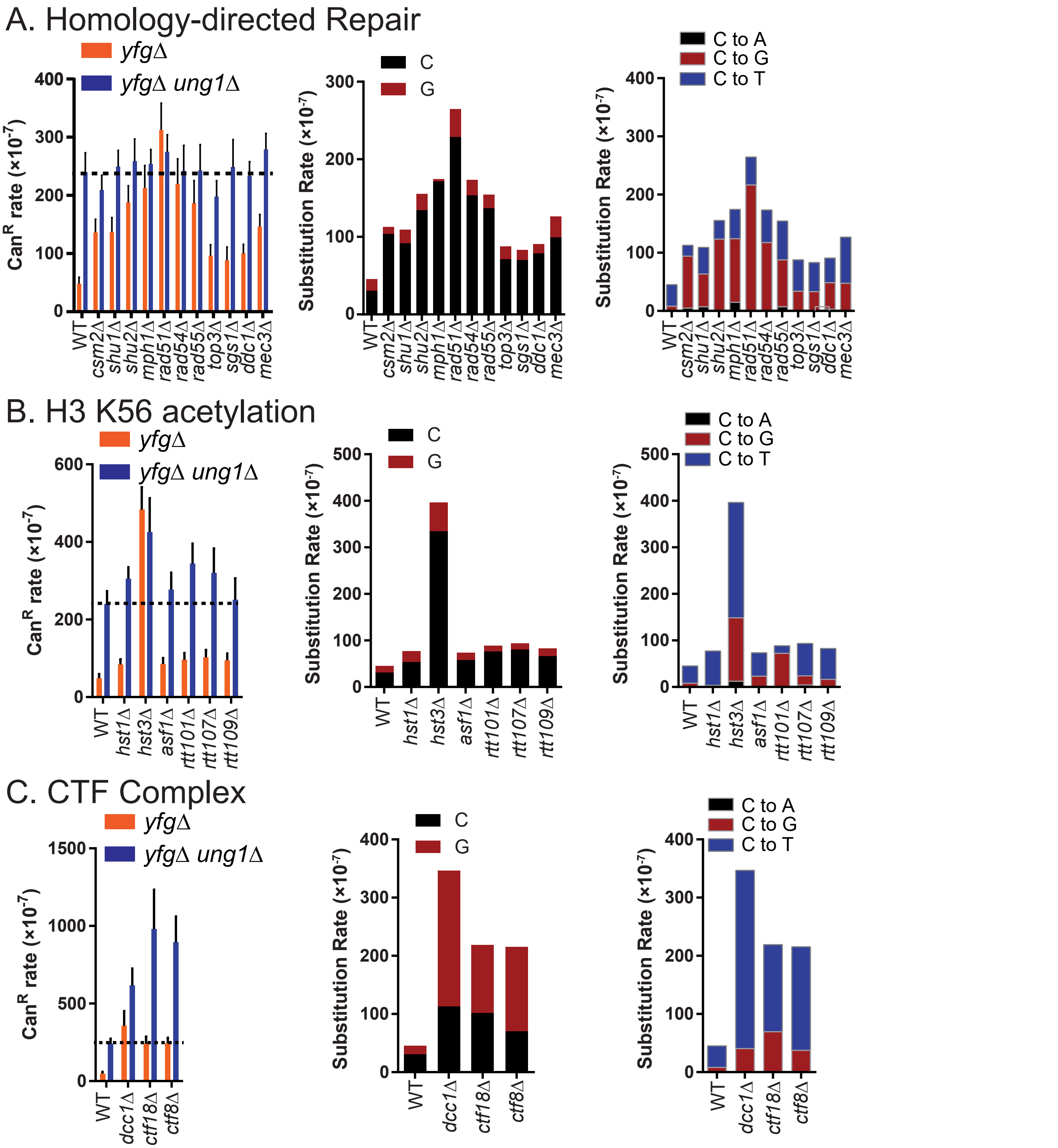

Supplemental Figure S2: Characteristics of APOBEC-induced mutagenesis in deletion strains with defective HR-directed repair, H3K56ac, and CTF18-RFC. (A) For yeast strains with deletions known to result in defective homology-directed repair, the A3B-induced Can^R rates for yeast strains with and without *UNG1*, rate of mutations at C and G nucleotides (stand-bias of A3B-induced mutations), and spectra of A3B-induced substitutions (indicative Rev1-dependent TLS usage) are compared. (B) For yeast strains with deletions of genes encoding proteins that modulate H3K56ac, the A3B-induced Can^R rates for yeast strains with and without *UNG1*, rate of mutations at C and G nucleotides (stand-bias of A3B-induced mutations), and spectra of A3B-induced substitutions (indicative Rev1-dependent TLS usage) are compared. (C) For yeast strains with deletions of genes encoding members of the CTF18-RFC complex, the A3B-induced Can^R rates for yeast strains with and without *UNG1*, rate of mutations at C and G nucleotides (stand-bias of A3B-induced mutations), and spectra of A3B-induced substitutions (indicative Rev1-dependent TLS usage) are compared.

Supplementary Figure S3

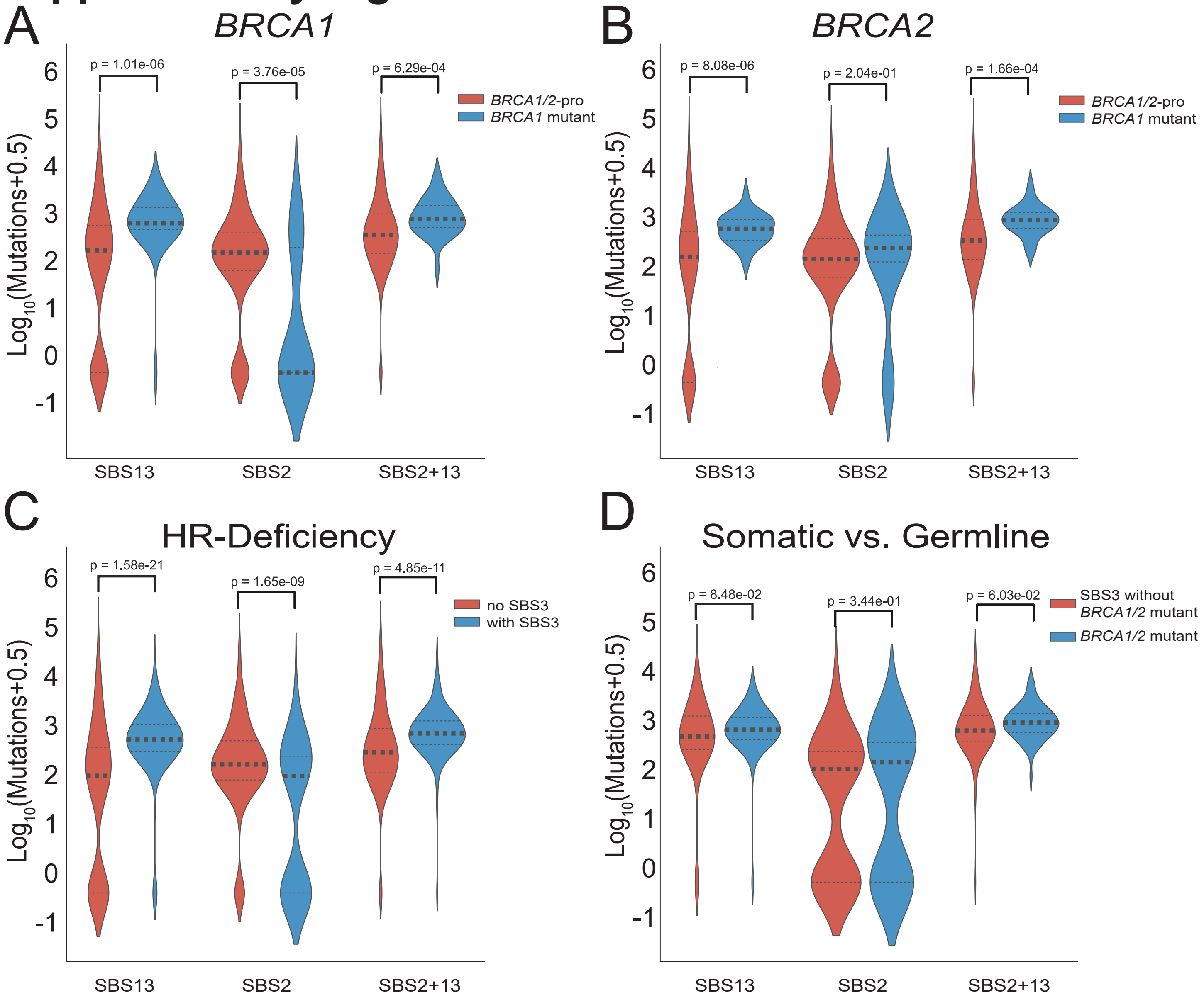

Supplementary Figure S3: Recombination deficient breast cancers have elevated APOBEC signature mutagenesis. Amounts of SBS2 and 13 in breast cancers with germline mutations in (A) *BRCA1* or (B) *BRCA2* compared to tumors without germline *BRCA1/2* mutations. (C) Amounts of SBS2 and 13 in breast cancers with and without SBS3 (indicative of HR-deficiency). (D) Amounts of SBS2 and 13 in breast cancers with SBS3 but no germline *BRCA1/2* mutations (i.e. somatically acquired HR-deficiency) compared to those in tumors with germline *BRCA1/2* mutations (i.e. germline HR-deficiency). Central dashed line indicates median values. P-values were calculated by Mann-Whitney U test.

Supplemental Figure S4

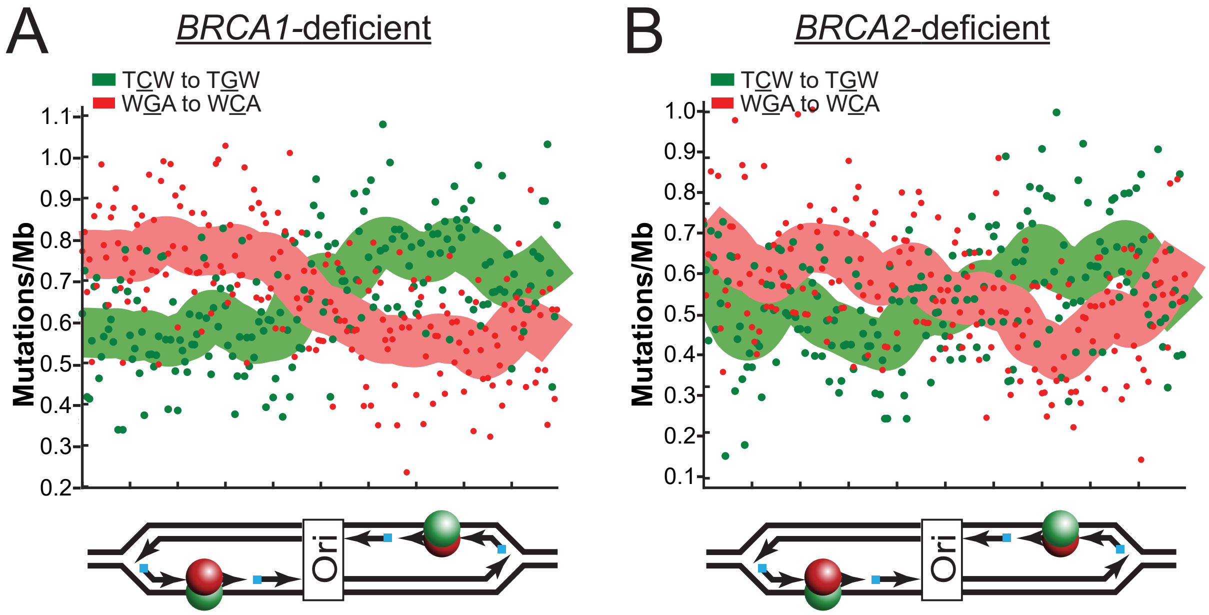

Supplemental Figure S4: Replication asymmetry of APOBEC-induced mutations in tumors with germline *BRCA1/2* mutations. Replication strand bias of SBS13 mutations in tumors containing germline (A) *BRCA1* or (B) *BRCA2* mutations.

LIST OF SUPPLEMENTAL TABLES

Supplemental Table S1: List of all screen hits and candidates

Supplemental Table S2: All mutation rates

Supplemental Table S3: RTq-PCR measurement of A3B expression in various yeast strains with single gene deletions

Supplemental Table S4: Numeric description of *CAN1* mutation spectra for each genotype

Supplemental Table S5: List of all *CAN1* mutations

Supplemental Table S6: Hierarchical clustering values

Supplemental Table S7: Strain genotypes and details on strain construction

Supplemental Table S8: List and description of oligonucleotides used

Supplemental Table S9: Enrichment component for GO analysis

Supplemental Table S10: Enrichment process for GO analysis
